# More procedures, more efficiency: optimizing operating room during the phase of learning curve—experience of first 100 robotic bariatric procedures in a single center

**DOI:** 10.1007/s11701-025-02396-0

**Published:** 2025-05-24

**Authors:** Zucchini Nicolas, Locci Eleonora, Moroni Enrico, Fantola Giovanni

**Affiliations:** 1 Department of Medical, Surgical and Health Sciences, University of Trieste, Trieste, Italy; 2 Metabolic and Obesity Surgery Unit, ARNAS G. Brotzu, Cagliari, Italy; 3 Department of Surgery, University of Cagliari, Azienda Ospedaliero-Universitaria, Presidio Policlinico di Monserrato, Monserrato, Italy

**Keywords:** Bariatric robotic surgery, Robotic surgery, Learning curve, Operating room efficiency

## Abstract

Robotic bariatric surgery (RBS) is increasingly adopted worldwide. This study aims to evaluate the implementation and evolution of RBS at a high volume center over five years, focusing on operative time (OT), operating room (OR) efficiency, and cost outcomes. A prospective analysis was conducted on patients undergoing elective RBS between July 2021 and March 2025 at ARNAS G. Brotzu, Cagliari. Metrics included OT, OR session time, and surgical volume. Variables analyzed included OT, OR session time, and surgical volume. Efficiency metrics such as overall OR efficiency, defined as OR session time/OT (Eff1), and robotic console utilization, defined as OR session time/console time (Eff2) were derived. Cost analysis incorporated OR activation time, surgeon and material costs. Statistical analyses included *t*-tests, Pearson’s correlation, and linear regression. 100 robotic-assisted procedures were recorded. Robotic adoption increased from 4.06% in 2021 to 38.98% in 2025. A learning curve (LC) was identified, with a significant OT reduction after the first 34 Roux-en-Y gastric bypass cases (*p* = 0.001). Full robotic manual anastomosis showed a notable cost decrease in later cases (*p* < 0.0001). Increased surgical volume correlated with both reduced OT (*r* = – 0.58) and improved Eff1 (*r* = – 0.49, *p* = 0.005). However, Eff2 changes were not statistically significant (*r* = – 0.31, *p* = 0.09), underscoring the need for team-wide coordination. RBS in high-volume centers enhance OR efficiency and cost-effectiveness over time. The LC, surgical volume, and institutional workflows were key factors in optimizing efficiency, highlighting the importance of a collective LC for the entire surgical team.

## Introduction

Minimally invasive surgery remains the cornerstone of bariatric procedures (BP). Beyond conventional laparoscopy, advances in surgical technology have led to the introduction and growing adoption of robotic platforms, aimed at enhancing precision, reducing complications, and improving recovery times. Most studies agree that both laparoscopic and robot-assisted BP are equally effective [1–3].

The use of robotic systems in BP has increased over the last two decades. Initially introduced to overcome the limitations of traditional laparoscopy—such as restricted dexterity and limited visualization—robotic platforms now offer enhanced precision, 3D visualization, and improved ergonomics. These advantages are particularly valuable in complex cases, including revisional surgeries and patients with BMI > 50 kg/m^2^ [4].

This rising trend in robotic bariatric surgery (RBS) has been observed globally. In the United States, data from the Metabolic and Bariatric Surgery Accreditation and Quality Improvement Program (MBSAQIP) database indicate a steady increase in robotic usage across all procedures between 2015 and 2020, with annual growth rates of 2.2% for sleeve gastrectomy, 2.0% for Roux en-Y Gastric Bypass (RYGB), and 2.4% for revisional surgery [5]. According to the International Federation for the Surgery of Obesity and Metabolic Disorders (IFSO) registry, robotic platforms are now used in nearly 30% of all BP in the US.

In Italy, data from the Italian Society for Obesity Surgery (SICOB) registry show a progressive increase in RBS procedures, from just 0.1–0.8% of total cases in 2014 to 1.7% and 3.7% in 2023 and 2024, respectively [4].

Despite the advantages associated with robotic technologies, laparoscopic surgery is still the standard of care in BP; while numerous studies have attempted to establish a clear superiority of robotic procedures over laparoscopy, no official consensus has been reached yet [6–8].

Nonetheless, the adoption of robotic platforms remains controversial, primarily due to concerns about higher healthcare costs compared to laparoscopy. Several studies report increased operative time (OT) with robotic surgery, particularly during the early phases of the learning curve (LC), potentially leading to operating room (OR) inefficiencies.

The LC is a critical phase in a surgeon's adoption of robotic surgery, as it directly impacts OTs, resource usage, and overall surgical efficiency.

The primary objective is to assess how OR occupancy time evolves during the surgeon’s LC and to identify strategies for optimizing efficiency. By understanding which factors influence OT, this study aims to streamline RBS workflows, reduce inefficiencies, and maintain high safety and outcome standards. These findings may provide valuable insights for both clinical and administrative decision-making to support optimal integration of robotic technology in BP.

## Materials and methods

This prospective study included consecutive patients who underwent elective RBS between the start of the robotic program in July 2021 and March 2025 at the Metabolic and Bariatric Surgery Unit of ARNAS G. Brotzu in Cagliari. Patients underwent one of three procedures: RYGB, One anastomosis gastric bypass (OAGB), or single-anastomosis sleeve jejunal bypass (redoSASJ) as a revision following sleeve gastrectomy.

The da Vinci Xi surgical system (Intuitive Surgical, Sunnyvale, CA, USA) was utilized for robotic-assisted surgery. It consists of a 3D vision system and EndoWrist instruments with 7 degrees of freedom that simulate dexterity and a range of movement, resulting in great precision and flexibility. All surgeries were conducted by a single skilled surgeon (G. F.) with more than 500 laparoscopic BP, primary and revisional, as consultant laparoscopic bariatric surgeon, actually director of Metabolic and Bariatric Surgery Unit, certified center of excellence by SICOB.

A descriptive statistical analysis was performed to summarize the characteristics of the study population. The dataset included the following variables: age, sex, weight, height, body mass index (BMI), comorbidities (i.e. hypertension, diabetes, dyslipidemia, gastroesophageal reflux disease, obstructive sleep apnea syndrome), and type of intervention (RYGB, OAGB, redoSASJ). For continuous variables (age, weight, height, and BMI), the mean, median, and standard deviation were calculated. For categorical variables (sex, comorbidities, type of intervention, and redo procedures), absolute frequencies and percentages were determined. The annual number of robotic procedures was compared to the total number of BP performed by the center during the same period. Results were organized and formatted for inclusion in the manuscript.

For RYGB, four categories were analyzed: Full Robotic manual anastomosis (FRMA), Full Robotic mechanical anastomosis (FRMeA), Hybrid manual anastomosis (HMA), and Hybrid mechanical anastomosis (HMeA). For OAGB, only FRMA and FRMeA were included. Due to the limited number of redoSASJ procedures, no distinctions based on technique were considered.

Various categories were analyzed to address the primary endpoint. OTs for each surgical procedure were recorded in minutes, including the time spent at the robotic console. Additionally, the total duration of each surgical session (i.e., total operating room occupancy time) was documented. Based on these values, efficiency metrics were derived: the ratio of effective surgical time to the total duration of the surgical session, and the ratio of time spent at the robotic console to the total duration of the surgical session, both expressed in minutes.

For each procedure, the OT, defined as the time in minutes from skin incision to skin closure, was recorded.

OR session time was defined as the total time (in minutes) the operating room was occupied for the daily procedures. These two values allowed for the derivation of efficiency metrics:

Overall OR Efficiency (Eff 1):

$$ Eff1 = \frac{Total\,OR\,session\,duration}{{Operative\,time}} $$

The ratio of effective OT to the total OR session time. A value closer to 1 indicates higher efficiency in OR utilization.

Robotic Console Utilization (Eff 2):

$$ Eff2 = \frac{Total\,OR\,session\,duration}{{Time\,at\,the\,robotic\,console}} $$

This metric evaluates the proportion of OR time dedicated to robotic surgery, which may influence efficiency.

The costs associated with each surgical session and procedure were calculated, in terms of theatre costs alone and not including length of stay, cost of care of the facility or eventual complications. These costs included the per-minute cost to activate the OR, the per-minute cost for each required surgeon, and the costs of materials used during the procedures. The per-minute cost for activating the operating room was set at €24, according to literature [9], including all the OR equipe, console and related. For Hybrid (H) procedures, an estimated additional cost of €50 per hour was included to account for the second consultant surgeon required in these procedures; contrarly in Full robotic (FR) procedures, the bedside assistant was a resident, which is not involved in hospital costs [10]. The costs of the FR and H approaches (FRMA, FRMeA, HMA, HMeA) were compared in terms of both efficiency and cost. Trends in the number of procedures performed monthly and annually were also analyzed, with correlations drawn between surgical volume, average procedure time, and Eff 1.

### Statistical analysis

To evaluate whether OT decreased significantly over the study period, the dataset was divided into two phases (early and late) for each procedure type. The early and late phases for RYGB and OAGB were defined as the first and last 34 and 14 cases, respectively. The threshold of 34 cases for RYGB was based on recent literature identifying approximately 30 cases as the inflection point in the learning curve for robotic RYGB, and was adjusted to match the distribution of our cohort [11]. For OAGB, a threshold of 14 cases was selected—approximately half—since these procedures began only after the RYGB learning curve had plateaued. An independent sample *t*-test was used, with statistical significance set at *p* < 0.05.

Differences in OT between RYGB and OAGB procedures were analyzed using *t*-tests on two separate groups. A subgroup analysis was also performed within the RYGB group to assess whether the type of approach (FR vs. H) or the anastomotic technique (manual vs. mechanical) significantly affected operative duration.

Additionally, annual trends in OT were analyzed. The mean OT was computed for each year and plotted against the number of annual procedures. A linear regression (LR) analysis was conducted to assess the presence of a trend, and the statistical significance of the correlation was evaluated using a *t*-test, with significance set at *p* < 0.05.

To evaluate the relationship between surgical volume and Eff 1, Pearson’s correlation coefficient (r) was calculated between the two of them. A negative correlation would suggest that increasing surgical volume is associated with an improved Eff 1 (i.e., Eff 1 approaching 1). The same correlation analysis, using both r and LR, was applied to OT and to Eff 2.

Cost differences were primarily analyzed by comparing FR and H procedures, calculating cost per minute for each approach and incorporating instrument costs. Secondarily, early and late phases of the robotic program were compared to evaluate whether costs decreased over time as the LC progressed.

Statistical tests, including independent sample *t*-tests and Pearson’s correlations, were conducted using built-in Excel functions and validated formulas. Regression analyses were supplemented by visual data representations including scatter plots and trend lines.

## Results

A total of 100 participants were included in the analysis. The mean age of the study population was 47.3 years (median: 48, Standard Deviation (SD): 10.5). The mean weight was 103.7 kg (median: 100.6, SD: 16.8), while the mean height was 159 cm (median: 159, SD: 6,7). The mean BMI was 40.8 kg/m^2^ (median: 40.4, SD: 5.3).

Regarding categorical variables, 4% of the participants were male and 96% were female; comorbidities affected 71% of the population. The distribution of intervention types was as follows: 68% underwent RYGB, 28% underwent OAGB, and 4% underwent redoSASJ (Table 1).

**Table 1:** Descriptive analysis

Population	Number	%
Male	4	4%
Female	96	96%
Comorbidities	71	71%
RYGB	68	68%
OAGB	28	28%
Redo SASJ	4	4%
Redo surgery	13	13%
Complications	4	4%

*RYGB* Roux en-Y gastric bypass; *OAGB* one anastomosis gastric bypass; *SASJ* single anastomosis sleeve jejunal bypass

**Fig. 1:**
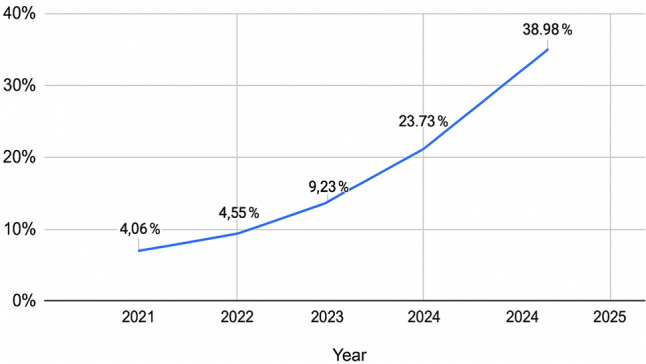
Percentage of robotic surgery of all yearly cases

Regarding the percentage of RBS per year in the same center, as demonstrated in Fig. 1, there was a constant increment, starting from 8 procedure out of 197 in 2021 (4.06%) to 23 procedures out of 59 (38.98%) in the first three months of 2025.

The mean OT per year demonstrated a progressive reduction from 2021 to 2025, reflecting a gain in robotic skills due to LC, but also the introduction of less complex procedures as OAGB. Comparison between the early and late phases of the study was not reliable due to differences in OT and could introduce bias.

OT was compared for each procedure (RYGB and OAGB). RYGB was divided into first 34 cases vs last 34 showing a significant reduction in OT (RYGB time 0 mean 172.27 min, SD 47.58 min vs RYGB time 1 mean 139.24 min, SD 33.66 min; *p* value = 0.002) (Table 2).

**Table 2:** Roux en-Y gastric bypass learning curve; Full robotic manual anastomosis vs hybrid manual anastomosis; One anastomosis gastric bypass learning curve

RYGB	First 34 cases	Last 34 cases	*p* value
Mean	172.27 min	138.33 min	
SD	47.58 min	33.77 min	
*T* test			0.001

FRMA vs HMA	FRMA	HMA	*p* value
Mean	149.60 min	160.97 min	
SD	46.73 min	46.53 min	
*T* test			0.296

OAGB	First 14 cases	Last 14 cases	*p* value
Mean	105.46 min	95.77 min	
SD	29.59 min	19.98 min	
*T* test			0.339

*SD* standard deviation; *FRMA* full-robotic manual anastomosis; *HMA* hybrid manual anastomosis

Since different techniques were performed, a subgroup analysis was conducted. In particular, it was not considered mechanical anastomosis, both FR and H, due to the low number of cases, but only considered differences in FRMA vs HMA. As reported in Table 2, there were differences in OT despite not statistically significant, showing that FR technique is quite shorter than H.

The OAGB group was divided into first 14 cases vs last 14. As reported in Table 2 there was no statistical significance, but it suggested an improvement in LC.

**Fig. 2:**
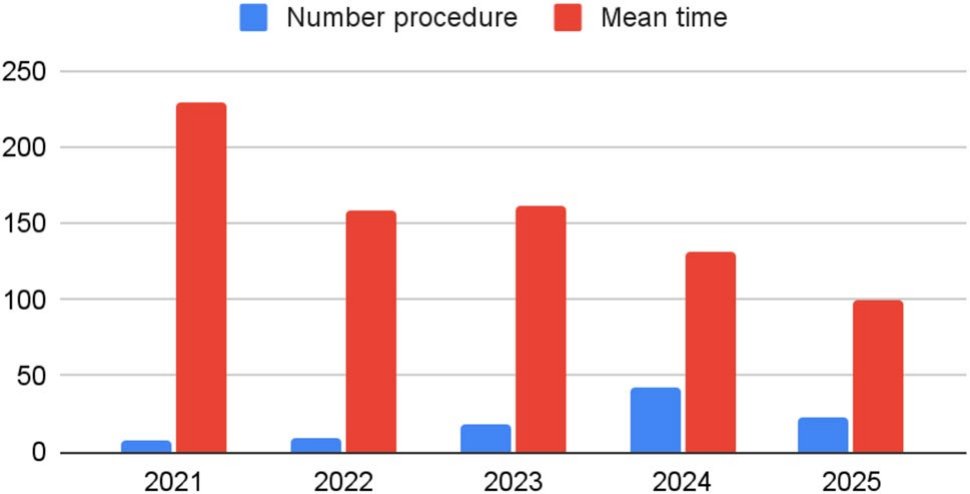
Number of robotic procedure per year vs mean operative time

Procedures time progressively improved over time in parallel with an increase in monthly and annual surgical volume. A negative *r* (*r* = – 0.58) was observed between the number of procedures performed per year and mean OT, indicating a moderate inverse relationship: as procedural volume increased, OT decreased (Fig. 2). This trend supports the hypothesis that cumulative surgical experience and improved workflow logistics significantly contribute to enhanced efficiency in the robotic setting. A negative *r* value, approaching – 1, suggests a stronger inverse correlation, and in this context, – 0.58 reflects a meaningful association between volume and performance.

**Fig. 3:**
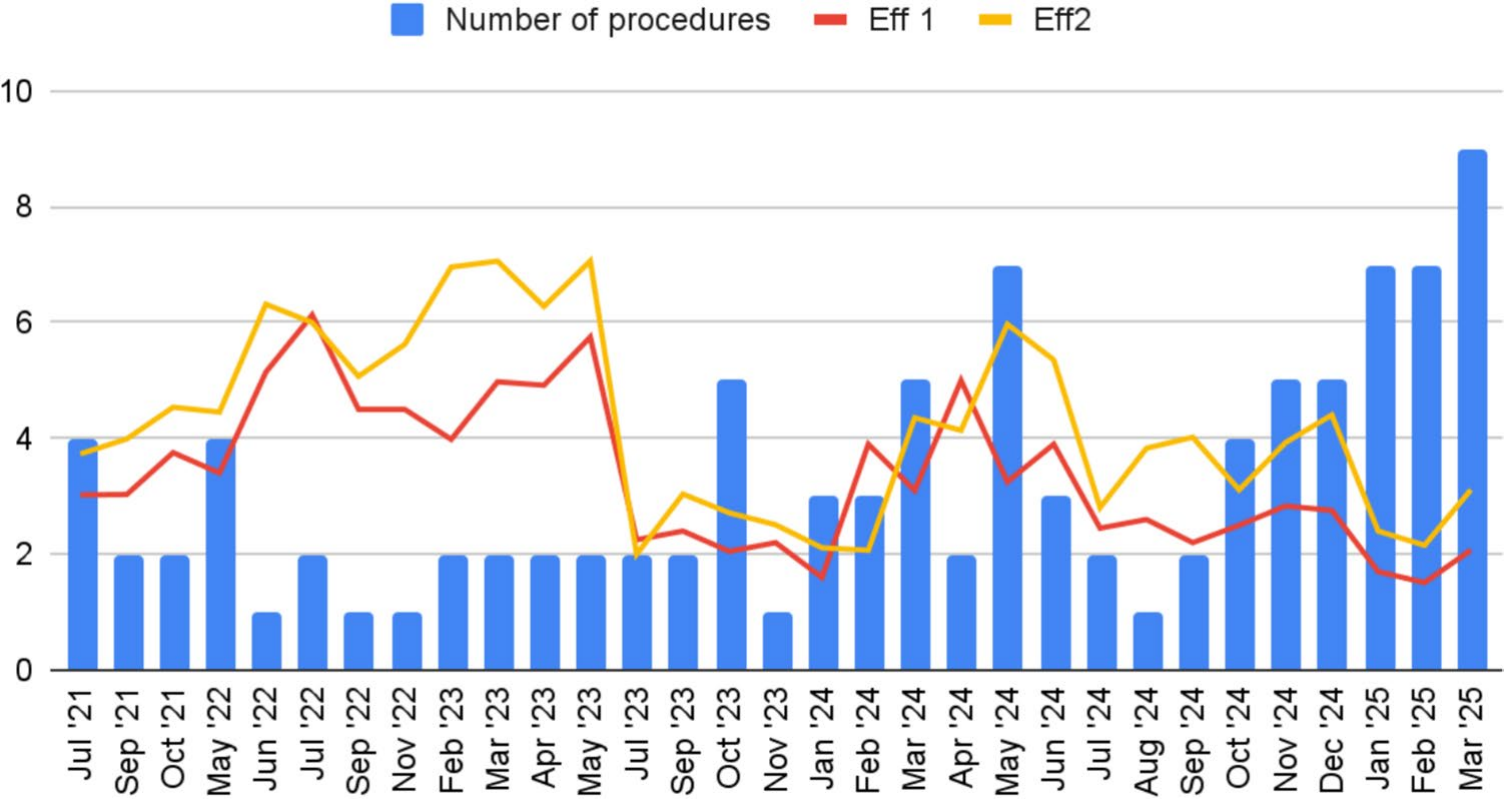
Number of robotic procedure per month vs Eff 1 & Eff 2

LR and r showed a negative correlation between monthly surgical volume and Eff 1, indicating that as the number of surgeries per month increased, Eff 1 values approached 1 (LR = − 0.81, *r* = − 0.49, *p* = 0.005). This analysis confirms that higher case volumes led to more efficient utilization of OR resources (Fig. 3).

Combining also Eff 2 in this analysis, r and LR for Eff 2, showed again a negative correlation between monthly surgical volume and Eff 2 even if without statistical significance (*r* = − 0.31, LR = − 0.41, *p* = 0.09) (Fig. 3). This can suggest that it is not directly correlated to the surgeon’s skills, but also to the OR staff's improvement in efficiency (i.e. docking, instrumental change, laparoscopic steps).

### Cost analysis

Cost evaluation showed no significant differences among FR versus H techniques for RYGB procedures, as showed in Table 3. Further analysis revealed no significant differences between FR and H techniques, even when comparing the first 12 and last 12 procedures (Table 3). Interestingly, when comparing the mean costs of the first 12 FR procedures with the last 12, there was a significant difference, confirming that as the number of procedures and efficiency increase, both OT and costs decrease.

**Table 3:** Cost analysis

RYGB	FRMA	HMA	*p* value
Mean cost	10942.68 €	10775.08 €	
SD	8473.91 €	7933.21 €	
*T* test			0.061

	FRMA first 12	HMA first 12	*p* value
Mean cost	11242.28 €	11589.06 €	
SD	45.10 €	50.64 €	
*T* test			1.000

	FRMA last 12	HMA last 12	*p* value
Mean cost	10642.28 €	10316.35 €	
SD	25.98 €	39.40 €	
*T* test			1.000

	FRMA first 12	FRMA last 12	*p* value
Mean cost	11242.28 €	10642.28 €	
SD	45.10 €	25.98 €	
*T* test			< 0.0001

## Discussion

The present study offers a comprehensive analysis of the implementation and evolution of RBS within a single medium-volume center over a five-year period. The findings demonstrate a progressive increase in the use of robotic-assisted procedures, consistent with international trends, and underline the positive impact of surgical volume on operative efficiency and cost optimization [12, 13].

One of the most notable observations was the steady rise in the percentage of RBS performed annually, from 4.06% in 2021 to nearly 39% in early 2025. This growth mirrors global patterns, likely driven by increasing surgeon confidence, institutional investment in robotic platforms, and evidence supporting the clinical and logistical benefits of robotic assistance. As technology evolves and institutional support for robotic platforms strengthens, the adoption of robotic techniques becomes more widespread, improving both outcomes and efficiency.

A significant LC effect was observed, particularly in RYGB procedures. When comparing the first 34 robotic RYGB cases with the last 34, a significant reduction in OT was noted (172.27 ± 47.58 vs 138.33 ± 33.77 min, *p* = 0.001). This supports the concept that procedural familiarity and team coordination improve over time, leading to shorter and more efficient surgeries [14]. The cumulative experience accumulated over multiple cases contributes to enhanced precision, improved surgical workflows, and reduced OT, which leads to multiple procedures in a single operative session [14]. The finding of no significant improvement in OT about OAGB may be attributed to the later introduction of OAGB into the robotic program—after substantial robotic experience had already been accumulated—resulting in relatively stable OTs even in the initial cases.

A gain of 10 min was reported for FR compared to the H approach without increased overall cost (*p* = 0.061). However, when comparing the first 12 FRMA procedures to the last 12, a significant cost reduction was observed (*p* < 0.0001), reinforcing the hypothesis that increased experience and improved workflow lead to greater cost-effectiveness, regardless of technique. This supports previous studies demonstrating that the LC and surgical volume can influence cost outcomes [15, 16]. Overall, the cost-effectiveness analysis suggests that although the initial investment in robotic technology is high, it may be offset by reduced OTs, improved Eff 1, depending mainly on increased number of procedures performed annually.

Moreover, our cost analysis confirms that increasing surgical volume leads to a reduction in cost per procedure; different authors suggested that robotic surgery, despite its initial high costs, becomes more cost-effective as experience is gained and resources are optimized [12, 17, 18]. However, some studies caution that cost differences between robotic techniques may not always be so evident, particularly in high-performance centers already operating at optimal efficiency. The correlation between surgical volume and efficiency was further supported by statistical analysis. A significant negative correlation was found between monthly surgical volume and OT (*r* = – 0.58), indicating that increasing the number of procedures contributes to decrease surgical time. This aligns with previous research showing that increased surgical volume is associated with reduced OT due to improved team coordination, quicker decision-making, and reduced time spent troubleshooting technical challenges [18, 19].

Similarly, Eff 1 showed a statistically significant improvement as monthly case volume of same speciality (i.e. bariatrics) increased (*r* = – 0.49, LR = – 0.81, *p* = 0.005), reinforcing how increased case volumes not only refine individual surgical proficiency but also contribute to enhanced OR logistics. This includes more individual surgeon efficiency, improved turnover management, and better coordination among OR team—key elements in optimizing the use of operative sessions and institutional resources. Although the correlation with Eff 2 was weaker and not statistically significant (*r* = – 0.31, *p* = 0.09), the trend suggests a wider improvements in non-console activities, such as docking, patient positioning, and laparoscopic steps. Notably, Eff 1 captures the overall surgical efficiency, while Eff 2 isolates the robotic console time from the total OR session. The discrepancy between these metrics can be interpreted as a surrogate marker for non-console OR logistics. The relative improvement in Eff 1—despite a stable Eff 2—indicates that gains in efficiency may be predominantly driven from better OR management, not just from improved console performance. As reported by Giedelman et al. cohesive teamwork, clear intraoperative communication, and systematic OR planning are central to enhancing efficiency, reducing delays, and lowering procedural costs [20]. Thus, while case volume is important, it must be accompanied by internal process optimization and effective multidisciplinary collaboration to fully realize the benefits of RBS. As highlighted also by Reddy et al. increasing surgical volume alone is not sufficient; optimizing internal processes and enhancing teamwork are equally crucial for improving operating room efficiency [21].

Previous authors, such as Giudeicelli et al. have proposed global benchmarks for robotic bariatric surgery, including an OT of less than 162 min for RYGB, an overall complication rate below 11.8%, and a hospital stay of less than two days in selected low-risk patients [22]. Our center required approximately 35 procedures to consistently remain below the OT benchmark, which supports recent findings on the learning curve in robotic bariatric surgery [12].

Nevertheless, this study has some limitations. First, the study did not include a detailed analysis of mechanical anastomosis (both FR and H), due to the limited number of such cases. This restricts our ability to assess whether anastomotic technique might influence OT, efficiency or costs in more complex subgroups. Second, the single-center design may introduce selection and institutional biases. Furthermore, the inclusion of different procedures over the study period—each with its own complexity and LC—poses an inherent challenge to direct comparisons. Future studies should aim to address these limitations by including a larger cohort and adopting a multicenter design to enhance the external validity of the findings.

## Conclusion

In conclusion, this study offers a novel, system-oriented definition of efficiency in RBS, demonstrating that improvements in OT and cost-effectiveness are driven by the optimization of the entire OR team. Implemented in a medium-volume and structured setting, RBS correlated with increased surgical volume and team experience.

By extending the definition of efficiency to embrace the entire OR team, this study proposes an expanded concept of the OR LC—one that applies not only to the primary surgeon, but to the full operative team. In particular, the refinement of non-technical skills, coordination, and role-specific performance becomes as crucial as technical capability at the console in driving operative efficiency. As such, robotic platforms represent a valuable strategic investment not just for surgical innovation, but for institutional transformation of the operative workflow.

The study supports the integration of RBS within high-performing systems where volume, coordination, and structure converge to enable sustainable improvements in efficiency, resource use, and surgical outcomes.

## Data Availability

No datasets were generated or analysed during the current study.
