# Correction: More procedures, more efficiency: optimizing operating room during the phase of learning curve—experience of first 100 robotic bariatric procedures in a single center

**DOI:** 10.1007/s11701-025-03042-5

**Published:** 2025-12-18

**Authors:** Nicolas Zucchini, Eleonora Locci, Enrico Moroni, Giovanni Fantola

**Affiliations:** 1 Department of Medical, Surgical and Health Sciences, University of Trieste, Trieste, Italy; 2 Metabolic and Obesity Surgery Unit, ARNAS G. Brotzu, Cagliari, Italy; 3 Department of Surgery, University of Cagliari, Azienda Ospedaliero-Universitaria, Presidio Policlinico di Monserrato, Monserrato, Italy

Correction: Journal of Robotic Surgery (2025) 19:233

10.1007/s11701-025-02396-0.

In the original version of the article, the given and family names of the authors were incorrectly structured but should have been

Given Name: Nicolas

Family Name: Zucchini

Given Name: Eleonora

Family Name: Locci

Given Name: Enrico

Family Name: Moroni

Given Name: Giovanni

Family Name: Fantola

